# Classification and coding of rare diseases: overview of where we stand, rationale, why it matters and what it can change

**DOI:** 10.1186/1750-1172-7-S2-A10

**Published:** 2012-11-22

**Authors:** Peter N Robinson

**Affiliations:** 1 Institut für Medizinische Genetik und Humangenetik, Charité - Universitätsmedizin Berlin, Augustenburger Platz 1, 13353 Berlin, Germany

## 

Carl Linnaeus published one of the most important early disease classifications in 1759, classifying a total of 325 diseases into 11 classes and 37 orders. This work, *Genera morborum*, provided a source of inspiration for a number of other classifications which paved the way for the classification of Bertillon in 1891 that subsequently became the first edition of the International Classification of Diseases (ICD). The latest edition of the ICD (ICD-10), includes nearly 500 rare diseases, only about 240 of which have a specific ICD code. With roughly 8000 named RDs and at least 100 new RDs characterized yearly, this means that less than 3% of RDs have codes in the ICD-10. Correspondingly, rare diseases have been largely invisible in national mortality and morbidity statistics, and policy makers have tended to allocate much fewer resources for research and clinical care in the field of rare diseases than might be expected given their overall prevalence of at least 5% of the population.

The new edition of the ICD (ICD-11, which is due by 2015) offers an opportunity to address these shortcomings. A Topic Advisory Group on Rare Diseases chaired by Ségolène Aymé has been coordinating efforts to create a comprehensive classification (nosology) of rare diseases for the new ICD. The classification is to follow a primarily clinical approach, and a polyhierarchy approach is used to include rare diseases affecting several body systems are included in each relevant chapter.

Phenotype ontologies such as the Human Phenotype Ontology complement disease classifications by providing a tool to describe and analyze the spectrum of signs, symptoms, and other abnormalities that people with the disease in question may display. A large number of different vocabularies and ontologies for human phenotype have been developed for different goals and users, but it will be essential to improve interoperability between these terminologies in the future in order to make maximum use of all available resources.

Classifications, ontologies, and other computational resources for human disease and phenotype will allow more accurate statistics about prevalence of rare diseases, better allocation of health care resources, and an improved ability to perform computational analysis of human disease manifestations for differential diagnosis and clinical decision support systems. Additionally, they will provide a basis for deep phenotype analysis to characterize the natural history of rare diseases and to discover clinically actionable complications and risks.

